# Emergence of novel ST1299 *vanA* lineages as possible cause for the striking rise of vancomycin resistance among invasive strains of *Enterococcus faecium* at a German university hospital

**DOI:** 10.1128/spectrum.02962-23

**Published:** 2023-10-31

**Authors:** Giuseppe Valenza, David Eisenberger, Sven Voigtländer, Rayya Alsalameh, Roman Gerlach, Sonja Koch, Bernd Kunz, Jürgen Held, Christian Bogdan

**Affiliations:** 1 Mikrobiologisches Institut – Klinische Mikrobiologie, Immunologie und Hygiene, Universitätsklinikum Erlangen, Friedrich-Alexander-Universität (FAU) Erlangen-Nürnberg, Erlangen, Germany; 2 Bayerisches Landesamt für Gesundheit und Lebensmittelsicherheit, Erlangen, Germany; 3 Apotheke des Universitätsklinikums Erlangen, Erlangen, Germany; University of Guelph College of Biological Science, Guelph, Ontario, Canada

**Keywords:** *Enterococcus faecium*, vancomycin resistance, bloodstream Infection, core genome multilocus sequence typing, ST1299 *vanA*

## Abstract

Since 2019, we have observed a remarkable rise of vancomycin resistance among invasive strains of *Enterococcus (E.) faecium* at the University Hospital Erlangen (UKER), a tertiary-care hospital in Germany. Here, we analyzed the genetic diversity of *E. faecium* isolated from blood cultures of in-patients by core genome multilocus sequence typing to see whether the occurrence of vancomycin resistance was associated with the spread of new clonal lineages. Between January and December 2022, 37 consecutive non-duplicate isolates were included in this study. Resistance to vancomycin was detected in 15 isolates (40.5%). The affected patients had a median age of 65 years, and 26 of them (70.3%) were males. Patients with bloodstream infection caused by vancomycin-resistant *E. faecium* (VREfm) or vancomycin-susceptible *E. faecium* (VSEfm) showed a similar in-hospital mortality rate (33.3% vs 27.3%, *P* = 0.692), indicating that VREfm were not more virulent than VSEfm. In contrast, the genetic diversity was higher in VSEfm (15 clonal lineages) than in VREfm isolates (seven clonal lineages). The majority of VREfm isolates (73.2%) belonged to only three clonal lineages: ST117/CT71 *vanB* (*n* = 4), which is the most commonly detected vancomycin-resistant lineage in Germany, and two novel ST1299 *vanA* lineages classified as CT3109 (*n* = 4) and CT1903 (*n* = 3). We conclude that the high rate of vancomycin resistance among invasive *E. faecium* isolates at UKER could be associated with the emergence of novel ST1299 *vanA* lineages, which is likely to be relevant also for other hospitals and countries. Future studies will focus on the prevalence of ST1299 *vanA* in the general population.

**IMPORTANCE:**

The proportion of VREfm among all *Enterococcus faecium* isolated from blood cultures in German hospitals has increased in the period 2015–2020 from 11.9% to 22.3% with a country-wide spread of the clonal lineage ST117/CT71 *vanB*. In this study, we provided useful information about the genetic diversity of invasive strains of *E. faecium*. Moreover, our findings confirm the nosocomial spread of novel ST1299 *vanA* lineages, which recently had a rapid expansion in Austria and the south-eastern part of Germany.

## OBSERVATION

According to data of the German antimicrobial resistance surveillance system (https://ars.rki.de), the proportion of vancomycin-resistant *E. faecium* (VREfm) among all *Enterococcus faecium* isolated from blood cultures has increased in the period 2015–2020 from 11.9% to 22.3% in German hospitals. This makes Germany one of the countries with a proportion of vancomycin resistance among invasive isolates of *E. faecium* above the European mean value of 16.8% (1). During the same period, a retrospective analysis of the genomes of all VREfm isolates collected within a German-wide study revealed a shift from *vanA* to *vanB* resistance and a prevalence of specific sequence types (STs) such as ST117 (2). In addition, an analysis of the genetic diversity performed by core genome multilocus sequence typing (cgMLST) at the German Reference Center for Staphylococci and Enterococci showed a country-wide spread of the clonal lineage ST117/CT71 *vanB*, with a proportion of 37% among 141 VREfm isolates from blood cultures in the year 2020 (3, 4).

The University Hospital Erlangen (UKER) is a tertiary-care hospital in Bavaria, Germany, with a capacity of approximately 1,400 beds. Until 2018, the rate of vancomycin resistance in *E. faecium* isolates from blood cultures of in-patients treated at UKER amounted to 9.8%. Since 2019, however, the proportion of VREfm has constantly been above 40% (2019: 46.3%; 2020: 41.9%; and 2021: 40.7%). Based on these observations, we analyzed all non-duplicate *E. faecium* strains isolated from blood cultures of UKER in-patients between January and December 2022 by cgMLST to see whether the occurrence of vancomycin resistance was associated with the spread of new clonal lineages.

Identification of isolates to the species level was performed using matrix-assisted laser desorption/ionization time-of-flight (MALDI-TOF) technology (Brucker Daltonik GmbH, Bremen, Germany). Antimicrobial susceptibility testing was carried out using VITEK 2 AST-P616 Cards (bioMérieux, Marcy-l’Étoile France) and MIC Test Strips (Liofilchem srl, Roseto degli Abruzzi, Italy). The results of antimicrobial susceptibility testing were interpreted according to European Committee on Antimicrobial Susceptibility Testing (EUCAST) breakpoints (eucast: Clinical breakpoints and dosing of antibiotics). Library preparation was performed using a Nextera XT DNA Library Prep Kit and a Nextera XT Index Kit (Illumina, San Diego, CA, USA). Libraries were quantified using an Agilent High Sensitivity DNA Kit (Agilent Technologies, Waldbronn, Germany) on a 2100 Bioanalyzer Instrument (Agilent Technologies, Santa Clara, CA, USA). On an Illumina MiniSeq system, 2× 150-bp paired-end reads were generated. Sequenced reads with a mean assembly coverage depth of 150 (range 68 to 304) were analyzed by the cgMLST scheme using SeqSphere+ software version 8.5.1 (Ridom GmbH, Münster, Germany) as previously described (5). Clonal lineages were defined as genotypes with a maximum difference of 20 alleles. To visualize the clonal relationship, a minimum spanning tree was generated using the abovementioned software. Furthermore, screening of genetic elements coding for antimicrobial resistance was performed by applying the publicly available database ResFinder 4.1. VirulenceFinder 2.0 and the virulence factor database (VFDB) were used to screen for putative virulence factors (6 – 8). In addition, *in silico* MLST was conducted using the PubMLST Database scheme implemented in SeqSphere+ software (9). All statistical analyses were performed using Stata SE (version 16.1).

A detailed characterization of the overall cohort of patients is presented in Table 1. At the time of the detection of *E. faecium*, 8 patients (21.6%) were treated in the hematology/oncology department (regular ward, *n* = 5; bone morrow transplant unit, *n* = 3), 7 patients (18.9%) in a surgical intensive care unit (ICU), 6 patients (16.2%) in two medical ICUs (nephrology, *n* = 3; pneumology/gastroenterology, *n* = 3), 5 patients (13.5%) in two medical normal wards (gastroenterology, *n* = 3; cardiology, *n* = 2), and 11 patients (29.7%) in other hospital wards (only one patient per ward). Resistance to vancomycin occurred in 15 *E. faecium* isolates of this study (40.5%). Among the vancomycin-resistant isolates, eight (53.3%) carried the *vanB*- and seven (46.7%) the *vanA* gene. Moreover, the following antimicrobial resistance rates were observed: erythromycin, 100%; ampicillin, 94.6%; ciprofloxacin, 94.6%; tetracycline, 48.6%; gentamicin (high-level resistance), 18.9%; daptomycin, 8.1%; linezolid, 0%; and tigecycline, 0%.

**TABLE 1:** Characteristics of patients with bloodstream infection due to *Enterococcus faecium* at the time of blood culture collection

Variables^*a*^	Total population (*n* = 37)	VREfm^*b*^ (*n* = 15)	VSEfm^*b*^ (*n* = 22)
Age, years, median (IQR)^*b*^	65.0 (48–73)	63.0 (48–72)	66.5 (43–75)
Sex, male (*n*, %)	26 (70.3)	12 (80.0)	14 (63.6)
ICU (*n*, %)^*b*^	13 (35.1)	7 (46.7)	6 (27.3)
Length of hospitalization, days, median (IQR)	32 (13–51)	47 (23–65)	21.5 (13-44)
Baseline comorbidities (*n*, %)			
Heart/cardiovascular disease	15 (40.5)	9 (60.0)	6 (27.3)
Solid malignancy	11 (29.7)	4 (26.7)	7 (31.8)
Hematological malignancy	9 (24.3)	4 (26.7)	5 (22.7)
Diabetes mellitus	4 (10.8)	2 (13.3)	2 (9.1)
Chronic kidney disease	4 (10.8)	1 (6.7)	3 (13.6)
Chronic liver disease	3 (8.1)	-	3 (13.6)
COVID-19	3 (8.1)	2 (13.3)	1 (4.5)
Chronic obstructive pulmonary disease	1 (2.7)	-	1 (4.5)
Devices (*n*, %)			
Central venous catheter	21 (56.8)	11 (73.3)	10 (45.4)
Urinary catheter	20 (54.1)	9 (60.0)	11 (50.0)
Central venous port system	6 (16.2)	3 (20.0)	3 (13.6)
Recent surgical procedure (*n*, %)	11 (29.7)	5 (33.3)	6 (27.3)
Hospital-acquired bloodstream infection (*n*, %)	26 (70.3)	13 (86.7)	13 (59.1)
Infection source (*n*, %)			
Urinary tract	7 (18.9)	2 (13.3)	5 (22.7)
Abdominal/gastrointestinal	6 (16.2)	2 (13.3)	4 (18.2)
Central catheter Infection	1 (2.7)	1 (6.7)	-
Unknown	23 (62.2)	10 (66.7)	13 (59.1)
In-hospital mortality (*n*, %)	11 (29.7)	5 (33.3)	6 (27.3)

^*a*^ Variables were not significantly different in patients with bloodstream infection caused by VREfm or VSEfm (*P* ≥ 0.05).

^*b*^ VREfm, vancomycin-resistant *Enterococcus faecium*; VSEfm, vancomycin-susceptible *Enterococcus faecium*; IQR, interquartile range; ICU, intensive care unit.

**Fig 1:**
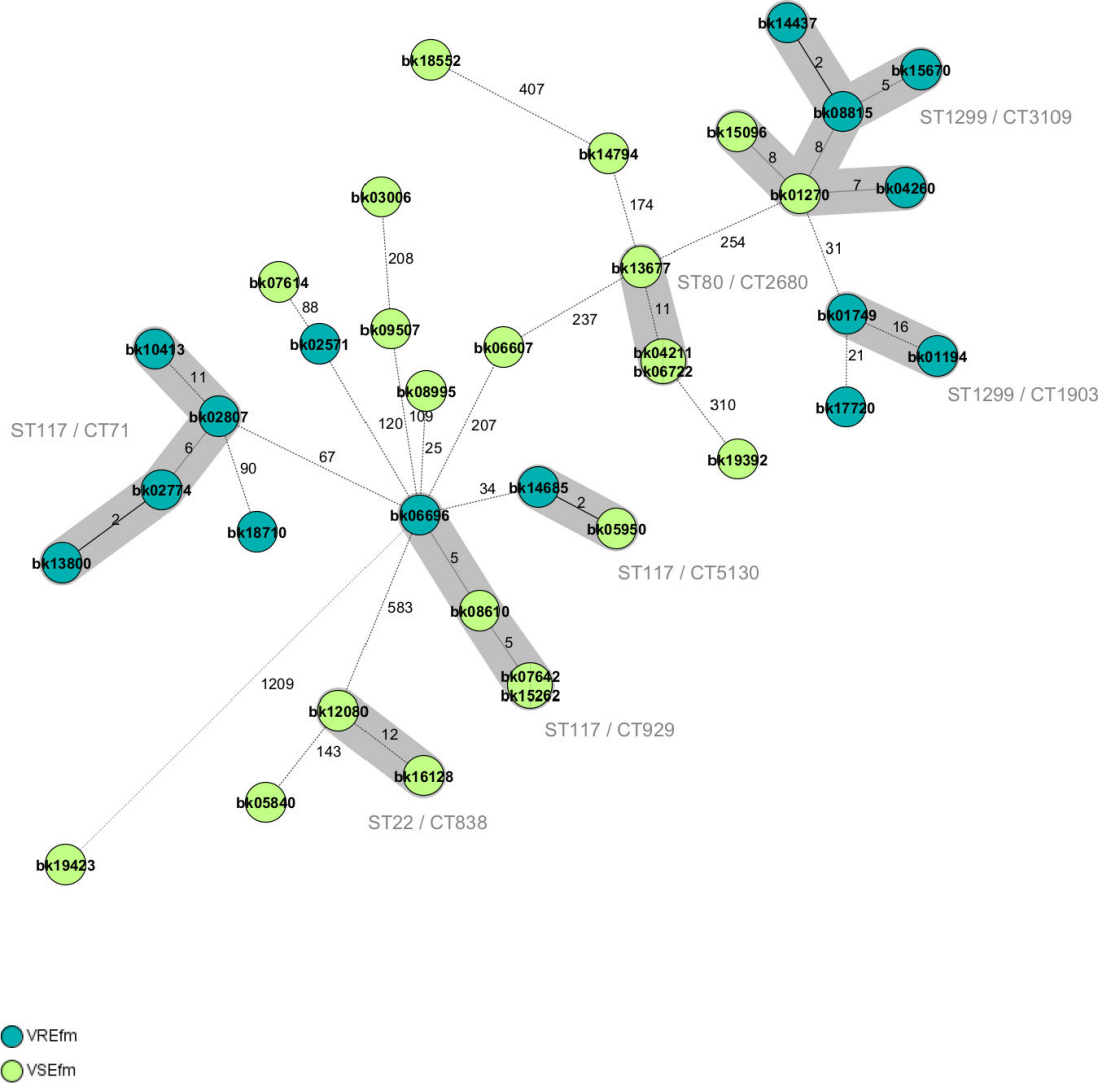
Minimum spanning tree of all *Enterococcus faecium* isolates (dark-green: VREfm—vancomycin resistant; light-green: VSEfm—vancomycin susceptible) of this study based on core genome multilocus sequence typing. Each circle represents isolates with an identical allelic profile based on up to 1,423 target genes present in the isolates with the “pairwise ignoring missing values” option turned on in the SeqSphere+ software during comparisons. The number in the circles represents the sample number. The number on connecting lines represents the number of alleles that differ between the connected genotypes. Genotypes belonging to the same clonal lineage (alleles difference ≤ 20) are gray shadowed.

*In silico* MLST revealed that *E. faecium* isolates of this study belonged to seven different STs. The most commonly detected STs were ST117 (*n* = 11), ST1299 (*n* = 9), and ST80 (*n* = 9). A significant difference in the distribution of the STs among the VREfm isolates compared with the vancomycin-susceptible *E. faecium* (VSEfm) isolates was observed (*P* = 0.022). ST1299 (*n* = 7) and ST117 (*n* = 6) were the most frequent STs in the VREfm group, whereas ST80 (*n* = 8) and ST117 (*n* = 5) were the most frequent STs in the VSEfm group. The genetic diversity as identified by cgMLST was higher in VSEfm (15 clonal lineages) than in VREfm isolates (seven clonal lineages). The majority of the VREfm belonged to three clonal lineages: ST117/CT71 *vanB* (*n* = 4), which is the most commonly detected VREfm lineage in Germany (3, 4), and two novel ST1299 *vanA* lineages, which were classified as CT3109 *vanA* (*n* = 4) and CT1903 *vanA* (*n* = 3) (Table 2; Fig. 1). The abovementioned ST1299 *vanA* lineages were first detected at the University Hospital Regensburg in the south-eastern part of Germany in 2018 and then have rapidly spread since April 2019 within the Regensburg County following an outbreak in a COVID-19 quarantine department of another hospital. Starting in 2021, ST1299/CT1903 *vanA* was also detected in Western Austria during outbreaks in various hospitals (10). In this study, isolates belonging to ST1299/CT3109 (VREfm, *n* = 4; VSEfm, *n* = 2) and ST1299/CT1903 (VREfm, *n* = 3) only differed by 31 alleles. Moreover, VREfm and VSEfm isolates belonging to ST1299/CT3109 were very closely related (alleles difference ≤ 20) as shown in Fig. 1. Interestingly, genetic elements coding for resistance to tetracycline such as *tet(M*) could be detected in six of seven VREfm belonging to ST1299 *vanA* (CT3109, *n* = 4; CT1903, *n* = 2). In contrast, none of the VREfm harboring the *vanB* gene (*n* = 8) carried *tet(M*). A genetic linkage between *tet(M*) and *vanA* on mobile genetic elements of ST1299 strains is therefore possible. Furthermore, the expression of *tet(M*) could play a role in the spread of ST1299 *vanA* lineages.

**TABLE 2:** Molecular characteristics of *Enterococcus faecium* isolates of this study

Variables	Total population^*a*^ (*n* = 37)	VREfm^*d*^ (*n* = 15)	VSEfm^*a*^ ^,*d*^ (*n* = 22)	*P*-value (<0.05)
Sequence types (*n*, %)				
ST117	11 (29.3)	6 (40.0)	5 (22.7)	0.022^*b*^
ST1299	9 (24.3)	7 (46.7)	2 (9.1)	
ST80	9 (24.3)	1 (6.7)	8 (36.4)	
ST22	3 (8.1)	-	3 (13.6)	
ST721	2 (5.4)	1 (6.7)	1 (4.5)	
ST116	1 (2.7)	-	1 (4.5)	
ST262	1 (2.7)	-	1 (4.5)	
Clonal lineages (*n*, %)^*c*^				
ST1299/CT3109	6 (16.2)	4 (*vanA*) (26.6)	2 (9.1)	Not determined
ST117/CT71	4 (10.8)	4 (*vanB*) (26.6)	-	
ST117/CT929	4 (10.8)	1 (*vanB*) (6.7)	3 (13.6)	
ST1299/CT1903	3 (8.1)	3 (*vanA*) (20.0)	-	
ST80/2680	3 (8.1)	-	3 (13.6)	
ST117/CT5130	2 (5.4)	1 (*vanB*) (6.7)	1 (4.5)	
ST22/838	2 (5.4)	-	2 (9.1)	
ST80/CT1065	1 (2.7)	1 (*vanB*) (6.7)	-	
ST721/CT6962	1 (2.7)	1 (*vanB*) (6.7)	-	
ST22/CT6963	1 (2.7)	-	1 (4.5)	
ST80/CT848	1 (2.7)	-	1 (4.5)	
ST80/CT2318	1 (2.7)	-	1 (4.5)	
ST80/CT2478	1 (2.7)	-	1 (4.5)	
ST80/CT6658	1 (2.7)	-	1 (4.5)	
ST80/CT6964	1 (2.7)	-	1 (4.5)	
ST116/ST7422	1 (2.7)	-	1 (4.5)	
ST117/CT5943	1 (2.7)	-	1 (4.5)	
ST262/CT2981	1 (2.7)	-	1 (4.5)	
ST721/CT1573	1 (2.7)	-	1 (4.5)	
Virulence factors (n, %)				
*acm*	37 (100.0)	15 (100.0)	22 (100.0)	
*bopD*	37 (100.0)	15 (100.0)	22 (100.0)	
*cpsA*	37 (100.0)	15 (100.0)	22 (100.0)	
*cpsB*	37 (100.0)	15 (100.0)	22 (100.0)	
*dll*	37 (100.0)	15 (100.0)	22 (100.0)	
*efaAfM*	37 (100.0)	15 (100.0)	22 (100.0)	
*fruA*	36 (97.3)	15 (100.0)	21 (95.4)	
*pilB*	36 (97.3)	15 (100.0)	21 (95.4)	
*srtC*	36 (97.3)	15 (100.0)	21 (95.4)	
*sagA*	35 (94.6)	14 (93.3)	21 (95.4)	
*sgrA*	33 (89.2)	15 (100.0)	18 (81.8)	
*scm*	27 (73.0)	8 (53.3)	19 (86.4)	
*pilA*	25 (67.6)	7 (46.7)	18 (81.8)	0.036
*capD*	23 (62.2)	8 (53.3)	15 (68.2)	
*ecbA*	16 (43.2)	8 (53.3)	8 (36.4)	
*hylEfm*	16 (43.2)	7 (46.7)	9 (40.9)	
*pilF*	14 (37.8)	1 (6.7)	13 (59.1)	0.002
*prpA*	14 (37.8)	5 (33.3)	9 (40.9)	

^*a*^ One isolate could not be assigned to a clonal lineage by core genome MLST.

^*b*^ *P*-value referred to the distribution of the sequence types in VREfm and VSEfm.

^*c*^ No clonal lineage occurred more than two times in a single ward.

^*d*^ Abbreviations: VREfm, vancomycin-resistant *Enterococcus faecium*; VSEfm, vancomycin-susceptible *Enterococcus faecium*.

Of note is the fact that none of the clonal lineages detected at UKER occurred more than twice in a single ward. Moreover, a comparison of the occurrence of putative virulence factor genes in VREfm and VSEfm revealed that VREfm were not more virulent than VSEfm (Table 2). These findings corroborate previous observations (11) and suggest that the spread of the VREfm at UKER is not associated with (i) a failure to implement infection control measures or (ii) the occurrence of hypervirulent strains. On the other hand, we cannot rule out a certain degree of bias, as the databases used in this study to analyze the occurrence of virulence factors only consider known virulence factors.

Our study also revealed that the in-hospital mortality rate was not significantly different in patients with BSI caused by VREfm or VSEfm. These results are consistent with a recent study on patients with BSI caused by *E. faecium* and *E. faecalis*, which showed that *E. faecium* was an independent risk factor for in-hospital mortality and vancomycin resistance did not further increase this risk (12).

Intestinal colonization is a prerequisite for VREfm infections and patient-to-patient transmission (13). A study on the fecal carriage of VREfm on hospital admission conducted at six German tertiary-care university hospitals between April 2014 and December 2018 showed increasing prevalence rates from 0.8% to 2.6%. In total, 78.5% of the VREfm were *vanB* positive and the predominant sequence type was ST117 (56.7%) (14). However, little is known about the prevalence of VREfm in German healthy individuals. The determination of the proportion of healthy individuals carrying VREfm in the general population of the city and county of Erlangen and the molecular characterization of the VREfm strains could help to better understand the relevance of the ST1299 *vanA* lineages detected at UKER.

In conclusion, the high rate of vancomycin resistance among invasive *E. faecium* isolates at UKER could be associated with the emergence of novel ST1299 *vanA* lineages, which is likely to be relevant also for other hospitals and countries. Future studies will focus on the prevalence of ST1299 *vanA* in the general population.

## Data Availability

The raw sequencing data were published at the European Nucleotide Archive (ENA), project number PRJEB64670.

## SUPPLEMENTAL MATERIAL

Table S1 spectrum.02962-23-s0001.xlsx Assembly of Illumina reads.

ASM does not own the copyrights to Supplemental Material that may be linked to, or accessed through, an article. The authors have granted ASM a non-exclusive, world-wide license to publish the Supplemental Material files. Please contact the corresponding author directly for reuse.
